# Babies under 1 year with atypical development: Perspectives for preventive individuation and treatment

**DOI:** 10.3389/fpsyg.2022.1016886

**Published:** 2022-11-17

**Authors:** Rosaria Ferrara, Leonardo Iovino, Magda Di Renzo, Pasquale Ricci

**Affiliations:** ^1^Department of Anatomy Histology, Legal Medicine and Orthopaedics, Sapienza University of Rome, Rome, Italy; ^2^Department of Economic and Legal Studies, “Parthenope” University of Naples, Naples, Italy; ^3^Institute of Orthophonology, Rome, Italy

**Keywords:** autism risk, developmental psychology, babies, early detection autism, pediatrics

## Abstract

A baby’s first year of life is a time of immense development and cerebral plasticity. Following today’s research and clinical observation, the period of the first year of life provides a new challenge inasmuch it is presently clear that it is possible to identify developmental anomalies in this window of time. Effecting early screening procedures could prove very useful, especially where we find genetic vulnerabilities in brothers and sisters of autistic subjects. Interventions of this kind, already practiced by some Public Health systems, can mean taking early action and primary protective measures with significant impacts not only on the subjects (babies and family members) concerned, but also on the public purse. It is, therefore, essential to provide for specific professionalized procedures for psychologists, pediatricians and neuropsychologists to be introduced through personnel highly specialized in interventions during the first year of life.

## Introduction

Autism spectrum disorders (ASDs) are neurodevelopmental disorders characterized by deficits in social communication, and restrictive, repetitive behavioral patterns emerging early in childhood development (American Psychiatric Association, 2013) which continue to dictate the subject’s behavior for the rest of his/her life (Ferrara et al., 2021). These children also show intensified emotional reactivity and difficulties in emotion regulation. Once considered a rare condition, ASD has gone through a shift in terms of diagnosis, with a significant increase over time (Ferrara et al., 2016).

Since developing sufficient understanding leading to ensuring a series of consultations to check on the weight, height, and vision of a baby, so we should also ensure the same awareness concerning a baby’s neuropsychological development and recommend mandatory consultations to assess the extent of development less obvious to the naked eye (comprehension, emotional, and social development etc) which is equally important. There are several examples of health systems that have been set up (Eapen, 2016) in this way, so that, based on genetic data, from the familial point of view, interventions are initiated for early screening of the brothers/sisters of an ASD patient. Data from genetic insights report an ASD development risk up to 14% (Chakrabarti and Fombonne, 2001; Lauritsen et al., 2005), however (Ozonoff et al., 2011) in the largest study on this issue, reports that 18.7% of children with at least 1 sibling Major with ASD developed the disorder. Ozonoff pointed out that the 2 strongest predictors of an ASD diagnosis were the sex of the child and the number of older siblings affected, with a 2.8-fold increase in the risk of ASD for male infants (25.9% of infants). High-risk males versus 9.6% of high-risk females and a further double increase in risk if there was more than one autistic older sibling.

It has now been scientifically demonstrated how a series of alarm bells can be observed in a subject’s first year of life (Baron-Cohen et al., 2000). The existence of a sociable smile, the avoidance of a direct gaze, motor development, just to give a couple of examples, are part of a behavior pattern which should receive attention in identifying typical or atypical development (Crais et al., 2006). Paying attention and early intervention in the course of atypical development has a significant positive impact in reducing the subject’s difficulties, the negative impact on families and lifetime costs to the individual, family, and society (Vivanti et al., 2014).

This article, therefore, aims to provide a short guide for psychologists who are involved in interventions by setting out the spectrum between typical and atypical development, and to open a debate on the possibility of intervention in this specific life span. Our intent is to give an overview of the skills underlying the emotional, cognitive, and emotional development of the child in the first year of age and to suggest a tool and a method of intervention that the scientific literature has already validated for children in the first year of life. Our contribution does not want and cannot be exhaustive: the development of the child in the first year of life is so complex that it would take a treatise to explain it. Therefore our synthesis effort certainly brings cuts and fails to explain all the interconnections necessary for a typical development. However, our intent is precisely to propose a contribution that, albeit in a synthetic way, gives an overview of how the typical development “should” be in the first year of life, what are the alarm bells to pay attention to and the possible questionnaire to be used in these cases and a possible scientifically validated intervention methodology. This contribution is intended to be a map of a territory still partly unexplored, for clinicians, doctors, and operators who operate or would like to operate in the sector.

Although intervention is important in atypical development, it is essential to keep in mind the nature of typical development in a baby, always remaining sensitive to the individuality of each subject who follows his/her own particular pattern, besides social and cultural conditioning (Karasik and Robinson, 2022).

In accordance with what other authors have already posited, “healthcare professionals need to be aware of 2 areas: (1) milestone competencies, for example, growth in the motor, cognitive, speech-language, and social–emotional domains, and (2) the eco-biological model of development, specifically, the interaction of environment and biology and their influence on development” (Malik and Marwaha, 2022). The rest of this article will hinge on the dichotomy between the extent of the baby’s range of capacities and the development model in interaction with the surrounding environment. At the end of our dissertation, we shall try to show, therefore, what the characteristics of atypical development could be (Khan and Leventhal, 2022).

### Baby as a competent being: Baby’s skills

In pursuance of the dichotomy mentioned in the last paragraph, we shall analyze, even only in a generic fashion, what the characteristics of healthy development are in those areas that we should be attentive to, and on the other hand, what is indicative of something that is not progressing as it should.

### Motor skills

Multiple researchers have proposed that early signs of ASD-related impairments may first manifest within the motor system and present as a motor delay (Ozonoff et al., 2008; Esposito et al., 2009; Flanagan et al., 2012). Motor skills development is part of those areas indicative of harmonious and healthy development. Motor skills are deliberate movements that a baby makes to achieve an objective. These fall into two major areas: fine motor skills and gross motor skills.

#### Fine motor skills

These are used for smaller actions like holding, picking things up between finger and thumb or wriggling toes in the sand. Also, the articulation of the tongue and mouth fall within the scope of fine motor skills. From birth, the baby shows signs of fine motor skills, by holding firmly mother’s finger with its hand. As it grows, such actions take on a more deliberate form: the baby begins to carry toys, grips the side of the cot to pull him/herself up, etc.

#### Gross motor skills

This is the co-ordination of large muscle groups like arms, legs, or the entire body. Babies learn from head to toe. Our upper body muscle control develops before our lower body muscle control. As babies grow, they first develop control of their neck (head control) and trunk (sitting balance) and then they learn to control their shoulders, then elbows, wrists, and finally, their fingers. The same goes for the lower body, starting at the hips first, then learning to control their legs, feet, and eventually toes. Bit by bit, as they grow, babies carry out these movements with greater purpose and they even become part, not only of motor movements, but even their communication.

#### Cognitive skills

Capacities in the cognitive area relate to the baby’s ability to understand and to interact with its surrounding environment, and these capabilities can be measured from the earliest months of life (O’Grady and Dusing, 2015). Interaction, play, imitation would be some of the areas to study to determine to what extent the baby allows itself to engage with its surrounding environment and its caregiver. The cognitive and thought capacities that develop in the first year of life are extremely important so that, at the end of the first year, the baby develops significant thought processes such as the concept of permanent objects, or rather the idea that a person/object continues to exist even though it cannot be seen.

#### Social emotional skills

During the first months of life, one can observe how the infant transforms a series of innate reflexes, like crying, smiling, or stretching out a limb, to deliberate actions relating to its environment and its caregiver (Mindell et al., 2017). Therefore, one can note how a series of reflexes, used to draw attention to its primary needs, become signals indicating desire for warmth and closeness. It should be recalled that already by about 7 months old, babies can marshal emotive impulses through auditory and visual channels, and, from 8 months, they begin to develop an understanding of other people’s actions and formulate deliberate behavioral responses in relation to others.

#### Speech language skills

The ability to learn language is wholly inborn from the very first hours of birth. In fact, babies can identify amongst all the sounds to which they are exposed, those which have linguistic value (Pathak and Sovani-Kelkar, 2022). Hearing continues to develop very rapidly over the early months after birth (Kalashnikova and Burnham, 2018; Barajas et al., 2021), indeed it is one of the first systems that even begins to develop in the fetus as we consider the major connection that exists between the baby while still in the belly and its mother’s voice (Moon, 2017) as being the preferred contact channel with the outside world in intrauterine life. Language development, like all the skills we observe, is not only dependent on the cognitive apparatus but also on facial and buccal musculature with a series of articulation functions (Potter et al., 2019). In the first year of life, therefore, the baby will develop from producing canonical babbling to producing a series of sounds composed of vowels and consonants which is a varied babbling, to being able to produce repetitive syllables with a variety of consonants and the first words linked primarily to its surroundings like “mamma,” “pappa” its favorite toys, “porridge.”

These different areas of development seem to be strongly interconnected functions for balanced development in each area leading to development in another area as opposed to damage to one specific skills set which then compromises development in other areas. Scientific literature has asserted that the establishment of certain prerequisites underpinning the skills listed above is important in the baby and two are especially extremely significant *viz*. joint attention (Gernsbacher et al., 2008) and imitation (Kuniyoshi, 2015). To explain joint attention, we need to imagine a triangle being in fact the ability of the infant to share its attention with other persons in a coordinated way. To give a simple example we could say to the infant “Where is papa?” or where is its favorite toy and we would expect the baby to look around the surrounding room and then turn its gaze back to us so that, in this case, a kind of attention triangle is formed which is fundamental to developing appreciation for the other skills areas, language (Charman, 2003) in particular. Through imitation, on the other hand, we can discern the infant’s capacity to copy what it sees and feels from adults or peers (Ingersoll, 2008). Imitation is an almost always impaired skill in autism, in different ways and degrees from child to child (Vivanti and Hamilton, 2014). Imitation allows, already from the face to face interaction with the caregiver in the first months of life, to learn through the vision of the surrounding world and attunement to it. Therefore, in autism, learning itself is damaged, also due to the lack or different quality of imitative skills. These skills are of such great importance that they are then included in intervention training for autistic children (Ingersoll, 2008).

One of the first to give a definition of imitation was Jean Piaget (Piaget, 1951) who studied this process, identifying certain developmental stages which cover reflex imitation, such as the new-born who reacts to crying by crying itself and smiling at a laugh, to deferred imitation which emerges after the age of two, by which time an infant can repeat an action at a later stage. Imitation is a form of adaptation through which we conform to the behavior of others, and it is very interesting to see how social psychology has used this concept (Asch, 1951). The ability to imitate is built on our mirror neurons (Rizzolatti et al., 2014), a classic example of this in understanding the action of these neurons is yawning which is very catching for someone who sees this action.

Another channel of communication is that of gestures (Ramos-Cabo et al., 2019) which appear to be preparatory to language development (Talbott et al., 2020). At about 9 months, performative gestures which the infant performs commence with a specific intention to communicate, and the typical example in this context would be clapping hands. Thereafter, at about the age of a year, a number of social gestures are used, which have an independent significance and emerge in social routine and play but can be used in different practical circumstances (pointing, waving bye-bye, blowing kisses, and so on) and also in symbolic contexts (Guevara et al., 2020).

### Co-regulation: Babies and environment

A fundamental concept of developmental psychology is that of co-regulation, beginning from Thompson’s definition (Thompson, 1994), developed during the ‘90s, where regulation is underlined as a necessary mechanism for processes of the infant’s internal and external worlds. Furthermore, the concept of co-regulation positions the body at the center of the learning process of the baby who, from the very first months of life, identifies a specific attachment figure based on care received. The relationship with this figure has well known consequences for the development of the baby (Bowlby, 1988; Draghi-Lorenz, 2001; Moretti and Peled, 2004). In their conversations parents and children modulate and adjust to each other’s voices and vocalizations in a variety of ways, thus anticipating and regulating the cycle of emotions. It is also through voice that the child activates and engages with close family, also constructing an internal representation of the most affectively and psychologically significant external impacts. Scientific contributions have shown this hypothesis to be a proven scientific construct are truly diverse, and we have not been able to cite them all, as space does not permit, but only a few. Damasio and Carvalho (2013) more recently, showed that the cognitive and emotional spheres are constructed in stages and through the intersection between the child’s internal and external worlds, however, the Self is also constructed in this way (Wallon, 1947). Gaddini (1969) pointed out that in the first weeks of life the baby’s perception of the outside world passes through a series of bodily modifications. The first behaviors that the child enacts are purely imitative. Therefore, imitation is a precursor of the processes of adaptation of the Ego and, in the first stage, is used by the child to perceive/know and in the second stage to be. It is through the tonic dialog (Toledo, 2009) between mother and child that the child will develop a series of skills. Emotional involvement in the dyad is also fundamental to the repetition of those simple gestures, such as the protrusion of the tongue, which were previously only seen through an intra-individual perspective (Di Renzo, 2017). But this *pas de deux*, as other studies on prenatal life point out, is nothing more than the continuation of a rhythm already established during pregnancy. Trevarthen (2001) coined the term sinus rhythm to refer to the dialog that exists between mother and child during pregnancy. Co-regulation is the ability that is expressed at the dyadic level between caregiver and child to regulate and treat their emotions, ideas, and behaviors. It is a prerequisite of a healthy emotional and cognitive organization that manifests itself with the child’s ability to express himself and adapt to the context, filtering, and organizing inputs from the internal and external world, thanks to the attunement with the caregiver. This process, as we have previously pointed out, has its roots in pregnancy. The scientific literature has clearly highlighted how much these abilities are damaged in autistic children (Jahromi et al., 2012; Chan et al., 2018). ASD children have fewer positive and more negative adaptive strategies when compared to non-ASD children. Such difficulties. Moreover, they are related to higher levels of emotional dysregulation (anxiety, depression) and behavioral problems related to aggression (Mazefsky et al., 2014).

### Alarm bells and/or predisposing factors for an atypical development

The first year of life is a time of development and learning about important matters which can give accurate signs of an atypical development trajectory, veering toward autism. Signs which may worry parents and/or pediatricians and specialists could relate to an inability to master a skill we have listed above, or with the emergence of repetitive and or excessive movements. This may sometimes also be encountered even in very young babies who are already presenting with motor stereotypies. We wish to summarize several signs which set off alarm bells for an atypical development which we can list as the following predisposing behaviors/conditions.

Brothers or sisters already on the autism spectrum: as has been shown in a wide range of scientific literature, where there is a brother or sister on the autism spectrum there is a possibility that he/she may also fall within the spectrum (Grønborg et al., 2013; Sandin et al., 2017). Recently it has been shown that the risk for unaffected families, as compared to the risk of occurrence of ASD is increased 8.4 times where there is an older brother with ASD and an increased risk of 17.4 times where there is an older brother with CA (childhood autism) (Hansen et al., 2019).

The presence of express atypical patterns of crying (higher fundamental frequency, shorter inter-bout pauses, fewer utterances) in response to social (Esposito et al., 2014) and non-social stressors (Sheinkopf et al., 2012). In addition, the cry of a baby with ASD is more difficult to decipher by a parent who finds it unexpected and inexplicable (Esposito and Venuti, 2008). It may also be very difficult to comfort these babies since cuddles and physical contact are not always well tolerated, and sometimes may be experienced as being invasive (Henderson, 2022).

Difficulties in gaze regulation and all such anticipatory gestures (Landa et al., 2007) which should develop within the first 6 months of life, through playing routines as well as through feeding (Boyd et al., 2010; Brisson et al., 2012) It is very important to follow the development of non-verbal forms of communication including fine motor acts such as pointing, as well as gross motor acts such as head turning for looking because gestural development is closely linked to language development (Iverson and Goldin-Meadow, 2005). Infants’ early rhythmic arm movements appeared to peak around the same time they began to babble (Erson and Wozniak, 2007). This relationship between early arm movements and babble onset was lacking in some infants who later developed ASDs as well as infrequent initiation of and response to joint attention cues of others (Yoder et al., 2009).

Motor-skills difficulties, delays in achieving motor milestones such as rolling, sitting, and crawling as well as atypical movement patterns such as movement asymmetries or abnormal reflexes (Bhat et al., 2012).

Cranial circumference, various scientific evidence has emphasized an accelerated increase in the circumference of the cranium taking place within the first 2 months of life with a significant surge in this increase from 3 to 5 months of life (Muratori et al., 2009).

Reciprocity, where reciprocity appears lacking in play, social, or sensory routines or simply during an interaction, such as a nappy change, or during mealtimes where the baby seems more interested in objects than in people and does not seem to understand the meaning of gestures (Schwartz et al., 2021).

### Psychological intervention for babies

An advancement of psychology in Italy could be about precisely this two questions: evaluation of the risk of autism or of atypical development and early intervention. Attention to these issues is spread unevenly in our country, despite the presence and effort of various health institutes that deal with them. One instrument useful in identifying the risk of developing a form of autism, or a similar atypia, within the first year of life, is as indicated on the grid. The so-called PREAUT (Olliac et al., 2017) grid can obtain a predictive VPP of 26% at 4 months which increases to 36% at 9 months. Furthermore, the PREAUT grid is repeatable at 4 and 9 months and the results are statistically correlated with CHAT (Allison et al., 2008) which is administered at 18 or 20 months (in particular, the key indicative, and practical items). By assessing children’s typical behaviors and play patterns, the CHAT scale is a screening tool for the likelihood of autism in children between the ages of 18 and 24 months (Robins and Dumont-Mathieu, 2006). It consists of five observation questions evaluating the child’s behavior and responses to examiner-initiated stimuli, as well as nine questions proposed to parents (items A), evaluating the child’s interest in social interactions, motor games, pretend play, pointing, and bringing an object to show it (B items: eye contact, pretend play, proto-declarative pointing, pointing comprehension, and construction of a tower of cubes). When a baby fails all five test items, they are deemed high risk. When an infant never points (proto-declarative score), neither their mother (A7) nor the examiner observes them, they are deemed “positive” with a medium risk (B4).

The PREAUT grid can be a useful guide for psychologists, neuropsychiatrists and other professionals involved in the early identification of atypical development. The PREAUT grid was created using clinical data from therapeutic treatment with at-risk children and observation of family films of children with autism. The researchers group proposed the theory that, in contrast to healthy children, children at risk of developing ASD may be hampered by their intrinsic drive to interact and be a source of joy for those they interact with. The PREAUT grid assesses a child’s capacity for synchronous and spontaneous play and joyous interactions. The PREAUT grid’s items, such as: Does the baby gaze at the examiner or mother (or her replacement) voluntarily or just when asked? have been developed to combat a lack of social initiative: in reality, the more a youngster participates in an interaction spontaneously and actively, the better is the Preaut score.

In France, were the grid is born, it is used by the doctor while the baby is having his or her routine appointment with the mother (or her replacement) at in the fourth and ninth month of baby.^1^

However, a psychological intervention at less than 1 year of life could be an intervention with a dual purpose. Thus, the psychologist may be able to facilitate co-regulation mechanisms between the baby and the caregiver as well as being able to work directly on the baby’s difficulties. It is certainly important that the psychologist be trained in specific methodologies for early intervention. Intervening as early as possible can improve the quality of life not only of the baby, but also of the family or caregivers. Studies have shown that interventions initiated after diagnosis within the first 3 years of life result in improved outcomes as compared to any interventions carried out subsequently (Vivanti et al., 2014). In any event, in the Italian context it is not easy to access the appropriate treatment when required during the window of neuronal plasticity. There is a long waiting list to access public services and not everyone is able to afford private treatment. Only a few have available financial resources and the majority will have to rely on public services. Nevertheless, it is vital to consider early intervention, but this also involves caregivers to provide them with techniques and strategies which they can use at home, as well as in nurseries attended by young children. In this way it would be advisable to train various persons, as well as therapists, who would be able to interact with the baby applying principles taught during dedicated training.

Early referral to successful therapy serves as a main means of preventing or delaying the full emergence of autism and the severe difficulties it is associated with. In order to have tested treatments available for identified newborns, early detection science demands that early treatment science evolve concurrently (Rogers et al., 2014). For precisely this reason, in this article we propose our intervention based on the ESDM (*Early Start Denver Model*) in various practical situations which will make training parents and school staff possible as well as providing for interventions for the child itself. ESDM is an intervention for effective (Dawson et al., 2010) developmental support for babies diagnosed with, or at risk of, ASD at the pre-school stage. As well as being a useful evaluation tool in the context of research (experimental efficacy), various studies in the Italian context have shown clinical efficacy (Devescovi et al., 2016; Colombi et al., 2018).

ESDM treatment is a comprehensive, manualized intercession program for children with ASD between the ages of 12 and 60 months (Dawson et al., 2010; Laister et al., 2021) is an approach based on techniques of Naturalistic Developmental Behavioral Intervention (NDBI).

Schreibman et al. (2015) this strategy envisions learning taking put inside naturalistic treatment settings that use the child’s inclinations and play exercises (Vivanti and Stahmer, 2020). Within the ESDM, there’s no outside reinforcement, but the joint and naturalistic exercises are intrinsically fulfilling for the child.

ESDM has also been scientifically validated with children under the age of 1 year who presented a series of behavioral atypia attributable to autism. Specifically, Rogers et al. (2014) described an ESDM intervention with seven symptomatic infants between the ages of 7 and 15 months with a 12-week low-intensity therapy. After treatment was over, parents kept up skills they had learned during the intervention and treated children at 36 months, had much lower rates of ASD than a similarly symptomatic group who did not enroll in the treatment study.

ESDM is shown to be a tool adaptable to needs, in as much as it can be used with a child, with a child and a parent (Parent-ESDM) (Rogers et al., 2012), in the school setting and electronically. In research by Dawson et al. (2010) it was shown how a group of 24 babies which received EDSM treatment for 20 h per week on a 1:1 basis in combination with methodologies for the parents, showed substantial improvement in most developmental areas 2 years after the intervention in comparison with children who had received other types of therapy of similar intensity. It is possible to involve parents effectively with ESDM therapy and similarly these strategies can be used at school through small-group interventions.

## Conclusion

The need for early tracking of the course of atypical development would appear clinically urgent and essential for research. It is now clear that early intervention brings about better outcomes because of the great cerebral plasticity of the first 2 years of life, leading to a better quality of life for the subject involved and his/her family. There are still only a few countries who engage in early screening measures. A country at the forefront of this is, as an example, Japan where there is a general health check-up at 18 and 36 months of age (Kamio et al., 2014). Annual statistical data by the Japanese Ministry of Health, Labor, and Welfare reports that more than 95% of all Japanese children at these ages are assessed in terms of their developmental levels at local public health centers every year. Another country benefiting from this approach is Australia: Government launched an initiative namely, the ‘My Child’s eHealth Record’ which recommends ongoing developmental checks (using Parent Evaluation of Developmental Surveillance) at 6, 12, and 18 months and 2, 3, and 4 years of age (Eapen, 2016).

For this reason, it is important to provide for training specifically for those who care for babies and those who wish to do so. Consultations for babies of less than a year old, presently take place without the application of the proper tools therefor: about 50–70% of primary healthcare workers do not use standardized tools for screening development during consultations with healthy infants, so that the accuracy of diagnoses is deficient when physicians base themselves solely on a clinical assessment (Miller et al., 2011). It would thus seem novel to apply this new and particular paradigm in which both the infants at risk and their families can receive psychological and/or specific specialist support without having to wait for an explicit diagnosis. Early identification means shifting from the ‘watch and wait’ approach to early referral and intervention of those identified to be at developmental risk and can substantially improve outcomes (Eapen, 2016).

## Data availability statement

The original contributions presented in the study are included in the article/supplementary material, further inquiries can be directed to the corresponding author.

## Author contributions

PR developed the theoretical formalism. All authors contributed to the article and approved the submitted version.

## Conflict of interest

The authors declare that the research was conducted in the absence of any commercial or financial relationships that could be construed as a potential conflict of interest.

## Publisher’s note

All claims expressed in this article are solely those of the authors and do not necessarily represent those of their affiliated organizations, or those of the publisher, the editors and the reviewers. Any product that may be evaluated in this article, or claim that may be made by its manufacturer, is not guaranteed or endorsed by the publisher.

## Footnotes

^1^ https://www.preaut.fr/dispositifs-experimentaux/la-recherche-preaut/recherche-preaut-princeps/#toggle-id-3

## Annexure 1: The PREAUT grid

**Table 1:**

1^st^ part of questionnaire (at 4 months if points = o < 3 move to 2nd part)	Yes	No
(1) Baby looks for caregiver		
(a) Spontaneously	4	0
(b) When spoken to (proto conversation)	1	0
(2) Baby directs its gaze to the mother (or substitute)		
(a) In the absence of any prompting, vocalization or movement while gazing intently	8	0
(b) When spoken to (proto conversation)	2	0
2^nd^ part of Questionnaire (only at 4 months if points = o < 3)	Yes	No
(1) In the absence of any stimulation by the mother (or substitute)		
(a) Gazes at mother (or substitute)	1	0
(b) Smiles at mother (or substitute)		
(c) Baby seeks an exchange of pleasure with the mother (or substitute) offering or extending feet or hands	4	0
(2) Following stimulation by the mother (or substitute)		
(a) Gazes at mother (or substitute)	1	0
(b) Smiles at mother (or substitute)	2	0
(c) Baby seeks an exchange of pleasure with the mother (or substitute) offering or extending feet or hands	4	0

A baby is considered at risk when the points are < o = 3 at 4 months or < o = 5 at 9 months.
